# Editorial: Cognitive Enhancement in Psychiatric Disorders

**DOI:** 10.3389/fpsyt.2019.00435

**Published:** 2019-06-19

**Authors:** Tomiki Sumiyoshi, Kenji Hashimoto

**Affiliations:** ^1^Department of Preventive Intervention for Psychiatric Disorders, National Institute of Mental Health, National Center of Neurology and Psychiatry, Tokyo, Japan; ^2^Division of Clinical Neuroscience, Chiba University Center for Forensic Mental Health, Chiba, Japan

**Keywords:** cognition, functional outcomes, therapeutics, assessment, biomarkers

Disturbances of various domains of cognitive function, e.g., several types of memory, executive function, attention, fluency, and attention/information processing, have been shown to provide a major determinant of outcome for patients with psychiatric conditions. Cognitive impairment is present not only in people with dementias but also in an array of diseases, including schizophrenia (with its prodromal stage), mood disorders, autism spectrum disorders, and eating disorders. This is in line with the Research Domain Criteria (RDoC) initiative proposed by National Institute of Mental Health in the USA, designating the Cognitive System as one of the functional domains whose impairment is pertinent to various operational diagnoses. Intervention into cognitive impairment has been recognized as a major goal of clinical practice, but much remains to be explored.

Articles in this *Topic* deal with genetic, molecular, imaging, physiological, psychological, and behavioral issues regarding the mechanisms, assessment, and treatment of cognitive disturbances in psychiatric illnesses. Twelve *original researches*, nine *reviews*, five *mini-reviews*, one *protocol*, and one *opinion* have been contributed by authors from eight countries in Europe, Middle East, Asia, North America, and South America.

To attain the aims of initiatives, such as RDoC, it is essential to elucidate the biological bases for abnormalities of specific cognitive problems. The first paper, Genetic Biomarkers on Age-Related Cognitive Decline by Lin et al. concerns the search for genetic biomarkers of cognitive aging. A review was provided on studies of candidate genes and genome-wide associations, as well as gene–environment interactions paradigms, which is relevant to the prevention and development of novel therapeutics. Also, Pu et al. in Right Frontotemporal Cortex Mediates the Relationship between Cognitive Insight and Subjective Quality of Life in Patients with Schizophrenia present data on cortical activities in the brain, as evaluated by near-infrared spectroscopy. Another powerful tool to assess brain functions *in vivo* is positron emission tomography (PET). Thus, Takano summarizes the role for receptor subtypes and transporters for dopamine, serotonin, and norepinephrine, as evaluated by PET, in cognitive disturbances of schizophrenia (Cognitive Function and Monoamine Neurotransmission in Schizophrenia: Evidence From Positron Emission Tomography Studies).

Neurophysiological evaluation in relation to behavioral changes associated with cognitive function has been an area of intensive research. Accordingly, Sueyoshi and Sumiyoshi provide a brief overview on this issue with particular focus on electrophysiological markers, including electroencephalogram (Electrophysiological Evidence in Schizophrenia in Relation to Treatment Response). For mood disorders, the study by Matsuo et al. (Sensorimotor Gating in Depressed and Euthymic Patients with Bipolar Disorder: Analysis on Prepulse Inhibition of Acoustic Startle Response Stratified by Gender and State) presents data of prepulse inhibition (PPI) of the acoustic startle reflex. These authors found PPI disruption in patients with bipolar disorder only if they are male and depressed, suggesting that this behavioral phenotype is both trait- and state-specific. The same group of investigators also report that *weak* handgrip strength and *high* body mass index are disadvantageous for cognitive function in patients with schizophrenia (Relationship of Handgrip Strength and Body Mass Index With Cognitive Function in Patients With Schizophrenia by Hidese et al.), providing a potential *physical index* for cognitive performance. Much attention has been paid to early intervention into cognitive and social outcomes in psychosis. This topic was reviewed in Early Intervention and a Direction of Novel Therapeutics for the Improvement of Functional Outcomes in Schizophrenia: A Selective Review by Kurachi et al., who summarized evidence for morphological changes in the brains of individuals with schizophrenia, and provides perspectives of novel treatments.

The implementation of reliable and valid assessment methods of cognition, capable of predicting social function, is one of the most important research topics. Specifically, Sumiyoshi et al. investigated high-level cognitive functions, using a text-mining technique, in individuals with schizophrenia, as reported in Semantic Memory Organization in Japanese Patients With Schizophrenia Examined With Category Fluency (by Sumiyoshi et al.). Comparisons of high-level cognitive function between subtypes of schizophrenia were attempted by Tyburski et al. (Neuropsychological Profile of Specific Executive Dysfunctions in Patients With Deficit and Non-deficit Schizophrenia), who observed the ability of performance on specific cognitive domains to predict long-term outcomes. For this clinical issue, Ohi et al. used the difference between premorbid and current IQs as a surrogate index of cognitive function in patients with schizophrenia (A Brief Assessment of Intelligence Decline in Schizophrenia as Represented by the Difference Between Current and Premorbid Intellectual Quotient).

Compared with schizophrenia, the degree of cognitive decline is milder in mood disorders, suggesting the need for using neuropsychological tests with greater sensitivity. Accordingly, Sumiyoshi et al. report the utility of the California Verbal Learning Test, with greater cognitive demands compared with other word list learning tests typically used for schizophrenia, in evaluating memory disturbances in patients with euthymic bipolar disorder (Verbal Memory Impairment in Patients with Subsyndromal Bipolar Disorder). Cognitive function also plays an important role in decision-making capacity of patients in the event of consenting to participate in clinical studies, receiving medical treatments, and so on. This critical issue is discussed by Sugawara et al. in Competence to Consent and Its Relationship With Cognitive Function in Patients With Schizophrenia. Overall, future research will be benefitted by incorporating objective markers to facilitate the understanding of the link between cognitive performance and real-world functional outcomes.

In an effort to develop effective therapeutics for cognitive impairment, translational approaches, i.e., bridging preclinical and clinical evidence, have been attempted. However, clinical trials of agents, produced through such approaches, have yielded negative results in most cases, indicating a need for further study. This challenge was summarized by Hsu et al. who provided a review of the literature on the efficacy of several drugs, i.e., putative “cognition enhancers” based on preclinical data, in treating cognitive impairment of psychiatric and neurological diseases (Medications Used for Cognitive Enhancement in Patients With Schizophrenia, Bipolar Disorder, Alzheimer’s Disease, and Parkinson’s Disease). The paucity of effective compounds to date is related to the initiative of the switch to other administration routes for the same drug, as reported by Hori et al. in Effects of Continuing Oral Risperidone vs. Switching from Risperidone to Risperidone Long-Acting Injection on Cognitive Function in Stable Schizophrenia Patients: A Pilot Study.

To overcome this situation, research into some of the novel agents is addressed. Thus, Guercio and Panizzutti provide a review of studies on the possible cognition-enhancing effect of D-serine, a co-agonist at N-methyl-D-aspartate receptors, and related compounds (Potential and Challenges for the Clinical Use of d-Serine as a Cognitive Enhancer). On the basis of animal data on spatial memory, Svoboda et al. discussed the role for subtypes of muscarinic acetylcholine receptors as a potential target for drug development (Drugs Interfering with Muscarinic Acetylcholine Receptors and Their Effects on Place Navigation). In addition to these agents, neurotrophic compounds, anti-inflammatory/oxidation agents, and particular nutrients may provide a novel candidate for pharmaco-therapeutics in this field. Also, whether prolonged administration of antipsychotic drugs is advantageous for improving cognition social function in schizophrenia and early psychoses has attracted attention. Accordingly, Omachi and Sumiyoshi examined results from relevant studies on this issue in Dose Reduction/Discontinuation of Antipsychotic Drugs in Psychosis; Effect on Cognition and Functional Outcomes, which should provide a clue to improving long-term consequences of quality of life for patients.

There is also a growing trend to develop non-pharmacologic therapeutics for ameliorating cognitive deficits in psychiatric illnesses. In particular, promising results have been reported for several types of cognitive remediation, or rehabilitation in schizophrenia and other diseases. Mogami contributed an *Opinion* on the Neuropsychological Educational Approach to Remediation (NEAR), one of the landmark methods of cognitive training (Cognitive Remediation for Schizophrenia with Focus on NEAR). Efficacy of this type of cognitive rehabilitation has been reported mainly for relatively young people with schizophrenia. With a well-controlled study design, Choi et al. found that disturbances of some of the key cognitive domains were alleviated also in older patients with chronic schizophrenia (Cognitive Remediation in Middle-Aged or Older Inpatients With Chronic Schizophrenia: A Randomized Controlled Trial in Korea). Several factors have been suggested to intervene the effect of cognitive remediation in schizophrenia. For example, Takeda et al. provided a review of the role for intrinsic motivation in optimizing the benefits of cognitive training, with reference to neurobiological substrates measured by brain imaging methods (Neural Correlates for Intrinsic Motivational Deficits of Schizophrenia; Implications for Therapeutics of Cognitive Impairment).

Recent efforts have been directed to boost the effect of cognitive rehabilitation with biological strategies, such as medications and non-invasive brain stimulation (neuromodulation). In this line, Harvey and Sand examined the current state of interventions combining cognitive and psychosocial treatments with pharmacological agents, such as stimulants, plasticity-inducing compounds, or attentional enhancers (Pharmacological Augmentation of Psychosocial and Remediation Training Efforts in Schizophrenia). For non-pharmacological approach, Jahshan et al. presented an update on co-treatment with physical exercise or transcranial direct current stimulation (tDCS), a type of neuromodulation (Enhancing Neuroplasticity to Augment Cognitive Remediation in Schizophrenia). Physical exercise is thought to stimulate neuroplasticity through the regulation of central growth factors, while the mechanisms of tDCS may involve long-term potentiation.

tDCS is the subject for a series of articles in this *Research Topic*, in accord with emerging evidence for the efficacy of neuromodulation in improving cognitive function. Thus, Narita et al. observed stimulation of the left prefrontal cortex with tDCS improved some domains of cognition, e.g., verbal memory, in patients with schizophrenia (Possible Facilitative Effects of Repeated Anodal Transcranial Direct Current Stimulation on Functional Outcome 1 Month Later in Schizophrenia: An Open Trial). Importantly, daily-living skills or “functional capacity” was also enhanced, providing the first report on the ability of tDCS to improve the higher-level functional outcome. These findings were based on an open-label trial, and the same group of investigators are conducting a confirmatory study with a more rigorous design (Effect of Transcranial Direct Current Stimulation on Functional Capacity in Schizophrenia: A Study Protocol for a Randomized Controlled Trial by Narita et al.). Several types of neuromodulation, such as tDCS, transcranial alternating current stimulation, and transcranial magnetic stimulation are also expected to enrich the treatment options for cognitive impairment in Alzheimer’s disease, as summarized by Chang et al. (Brain Stimulation in Alzheimer’s Disease).

Cognitive function and its dysregulation have been a topic of research on psychiatric conditions common in children and adolescents. Among the behavioral phenotypes typical of developmental disorders, sensory symptoms are included in the diagnostic criteria for autism. With a focus on acoustic startle response, Takahashi et al. observed that exaggerated acoustic reactivity was associated with skewness of locomotor activity in boys with autism spectrum disorders (Acoustic Hyper-Reactivity and Negatively Skewed Locomotor Activity in Children With Autism Spectrum Disorders: An Exploratory Study). In young women with attention-deficit/hyperactivity disorder (ADHD), gains of procedural memory consolidation were greater with the evening rather than morning training, unlike the case for individuals with typical development, as reported by Korman et al. (Procedural Memory Consolidation in Attention-Deficit/Hyperactivity Disorder Is Promoted by Scheduling of Practice to Evening Hours).

Impaired cognitive function has also been suggested to underline some aspects of the psychopathology of eating disorders. Thus, Tamiya et al. compared cognitive profiles between the restricting type and binge-eating/purging type of anorexia nervosa, and found significantly worse attention/vigilance for the latter type, which may be related to the higher mortality rate (Neurocognitive Impairments Are More Severe in the Binge-Eating/Purging Anorexia Nervosa Subtype Than in the Restricting Subtype).

These days, we occasionally receive a message “Tomiki (Kenji), an article you edited reached an impact milestone.” At the time of completion of this *Editorial*, the number of “views” of our *Topic* has reached almost 73,000, thanks greatly to the dedicated authors worldwide. This status assures us that our endeavor to “make a forum for researchers and clinicians interested in cognitive impairment of psychiatric illnesses” has been attained. This collaborative work accomplished by our colleagues will help facilitate the development of therapeutics of greater clinical value.

## Author Contributions

TS wrote the first draft of the manuscript, and KH provided opinions on it. Both authors read and approved the submitted version.

## Funding

This work was partly supported by Japan Society for the Promotion of Science (JSPS) KAKENHI No. 17K10321, Intramural Research Grant (29-1, 30-1, 30-8) for Neurological and Psychiatric Disorders of National Center of Neurology and Psychiatry (NCNP), and AMED under Grant Numbers 18dk0307069, 18dk0307081 and 19dm0107119h0003.

## Conflict of Interest Statement

The authors declare that the research was conducted in the absence of any commercial or financial relationships that could be construed as a potential conflict of interest. 

